# Smoking prevalence, reduction, and cessation during pregnancy and associated factors: a cross-sectional study in public maternities, Rio de Janeiro, Brazil

**DOI:** 10.1186/s12889-015-1737-y

**Published:** 2015-04-19

**Authors:** Pauline Lorena Kale, Sandra Costa Fonseca, Kátia Silveira da Silva, Penha Maria Mendes da Rocha, Rosana Garcia Silva, Alinne Christina Alves Pires, Maria de Lourdes Tavares Cavalcanti, Antonio Jose Leal Costa, Tania Zdenka Guillén de Torres

**Affiliations:** Departamento de Medicina Preventiva, Faculdade de Medicina, Universidade Federal do Rio de Janeiro (UFRJ), Avenida Brigadeiro Trompowski, Rio de Janeiro (Hospital Universitário Clementino Fraga Filho, 5º andar), RJ 21044-020 Brazil; Departamento de Epidemiologia e Bioestatística, Instituto de Saúde Coletiva, Universidade Federal Fluminense (ISC/UFF), Av. Marquês do Paraná, 303, 4º andar (Prédio anexo ao Hospital Universitário Antonio Pedro), Niterói, RJ 24130-210 Brazil; Núcleo de Epidemiologia, Instituto Fernandes Figueira, Fundação Oswaldo Cruz (IFF-FioCruz), Av. Rui Barbosa, 716 - Flamengo, Rio de Janeiro, RJ 22250-020 Brazil; Secretaria Municipal de Saúde e Defesa Civil do Rio de Janeiro (SMSDCRJ), Rua Afonso Cavalcanti, 455/sala 809, Cidade Nova, Rio de Janeiro, RJ CEP 20211-110 Brazil; Instituto de Estudos em Saúde Coletiva, Universidade Federal do Rio de Janeiro (IESC/UFRJ), Avenida Horácio Macedo S/N -– Cidade Universitária, Rio de Janeiro, RJ 21941-598 Brazil

**Keywords:** Smoking, Tobacco use cessation, Pregnancy, Health Inequalities

## Abstract

**Background:**

Smoking epidemic in Brazilian women has later onset, smaller magnitude, and slower decreasing trend, compared to men. Among pregnant women, smoking has an additional deleterious effect. The purpose of this study was to analyze smoking prevalence during pregnancy and associated factors, and to describe the frequency of smoking reduction and cessation in public maternities of Rio de Janeiro State, southeastern Brazil, in 2011.

**Methods:**

A cross-sectional study was conducted in two maternities located at public hospitals in two cities of the Rio de Janeiro state, Niterói (maternity A) and of Rio de Janeiro (maternity B). Data were gathered by interviews 12 hours after the delivery, and analyses of prenatal cards and medical records. Smoking prevalence according to maternal characteristics, adequacy of prenatal care, and proportions of smoking reduction and cessation during pregnancy were calculated. Factors associated to smoking during pregnancy were estimated by logistic regression analysis.

**Results:**

Smoking prevalence at maternity A (24.8%, 95% CI: 21.1-29.0) and maternity B (17.9%, 95% CI: 15.8-20.1) were high. Prevalence rates were greater in women aged 20-34 years, mainly without partner, multiparous and brown or black skin color. Low education (OR = 2.14, 95% CI 1.21, 3.79) and multiparity (OR = 3.48, 95% CI 1.78, 6.81), at maternity A; adolescence (OR = 0.44, 95% CI 0.26, 0.75), black skin color (OR = 1.71, 95% CI 1.06, 2.74), low education (OR = 1.61, 95% CI 1.08, 2.40), and multiparity (OR = 1.58, 95% CI 1.03, 2.44), at maternity B, were associated with smoking in multivariable analysis. Adequacy of prenatal care and smoking prevalence showed an inverse association. More than half of the smokers kept the smoking habits during pregnancy. Reduction occurred mainly between the 1^st^ and 2^nd^ trimesters of pregnancy.

**Conclusion:**

Smoking prevalence during pregnancy was higher for multiparous and less educated women. Population and individual strategies for smoking prevention and control must include actions specific for women, especially during the reproductive period.

## Background

A substantial decline in Brazilian smoking prevalence was detected between 1989 and 2008, especially in the 15-34 years age group, for those with higher education and among men [1]. Smoking prevalence among women aged 18 years or more in 2012 in Brazil’s capitals and in the Federal District was 9.2% [2]. A review of national surveys observed a 50% smoking decline among pregnant women from 1989 to 2008 [3]; albeit, the prevalence rate remains high in certain regions, around 20% [4]. Smoking epidemic in Brazilian women has later onset, smaller magnitude, and slower decreasing trend, compared to men. Among pregnant women, smoking has an additional deleterious effect [3,5,6]. Reduction and cessation of smoking habits during pregnancy have not been discussed in these articles.

Many negatives outcomes for smoking pregnant women have been observed, such as spontaneous abortion, ectopic pregnancy, infertility [3,7,8], gestational and post gestational complications [5], incidence and recurrence of births of small for gestational age [4,9], low birth weight, preterm birth [3,10], and risk factors for cardiovascular disease in young adults [11]. Smoking is the most important preventable risk factor and has greater impact concerning complications during pregnancy and labor [3,7].

Clinical trials showed evidence for promoting smoking cessation during pregnancy, asides reducing perinatal outcomes [12]. Results obtained from cost-benefit analyses in promoting smoking cessation during pregnancy were found to be better than in the population in general. This is partially explained due to the additional incentive concerning the child’s health [10]. The effectiveness of a smoking cessation program conducted in Denmark was similar between pregnant and no pregnant women, except for the subgroup of young smokers with disadvantages regarding schooling and employment. In such case, smoking cessation was greater among pregnant women in relation to nonpregnant ones [8].

Smoking habit and the difficulty to stop it may have features in common, among pregnant women, such as low education, low income, and late commencement of prenatal care [2,13].

The purpose of this study was to analyze smoking prevalence during pregnancy and associated factors and to describe smoking reduction and cessation in maternities of the national health care system (SUS) of Rio de Janeiro state, southeastern Brazil, in 2011.

## Methods

A cross-sectional study was conducted in two maternities of public hospitals of the national health care system (SUS) with higher frequency of liveborns in the Niterói (maternity A-MA) and Rio de Janeiro (maternity B-MB) cities of Rio de Janeiro state, southeastern, Brazil, between September and November of 2011. Both maternities are reference to low and high-risk pregnancy: MA is a regional reference and MB provides maternity care essentially to pregnant women resident in its neighborhood.

The present study results from the cooperation between southeastern Public Universities and Research Institution and was undertaken within the framework of the research projects “Study of perinatal and women mortalities in the metropolitan region of the State of Rio de Janeiro from 2006 to 2010” (Ministry of Health-MS/ Education Program in Work for Health - PET/Health Surveillance - Public Notice no. 7/2010), “Maternal and perinatal morbidity and mortality in the cities of Rio de Janeiro and Niterói: role of race, education, and social class in accessing health care services” (CNPQ - Public Notice 20/2010) and “The study of mother and child, an urgent need to meet the Millennium Development Goals” (FAPESP - Public Notice PPSUS/2009).

Between September and November of 2011, the maternities A and B were visited daily and all births were identified. Data were gathered 12 hours after delivery, from interviews to mothers, prenatal cards and medical records by trained undergraduate students from the health care area**.** All mothers of singleton liveborn babies were selected and those with missing information on smoking habits were excluded for the analysis.

The frequency and interruption of smoking habits from one month before pregnancy up to labor were evaluated by making five questions about the experience of smoking cigarettes, cigars, pipes or cigarillos during pregnancy, disregarding marijuana cigarettes, in the previous month to the pregnancy onset and for each trimester of pregnancy, based on National Longitudinal Survey of Children and Youth, 2008/2009 [14]. The smoking frequency classification (smoked daily/smoked less than daily/never smoked) didn’t consider the number of cigarettes smoked per day.

Smoking prevalence (at least one positive answer before or during pregnancy) was calculated according to the following variables: age group (10-19, 20-34 and 35 or older), self-reported skin color (white, brown – aggregating mulatto, and pardo, and black), partner (absence/presence), mother’s educational level in years (up to 4, 5-7, 8-11 and 12 or more), family monthly income *per capita* (or income, variable originally continuous, categorized according to the first three quartiles, in USD: up to $88.42, from $ 88.42 to $ 331.75, and ≥ $ 331.75), parity (primiparous/multiparous) and adequacy of prenatal care (Kotelchuck’s Adequacy of Prenatal Care Utilization Index) by maternity [15]. The maternal age group referred to the moment of the delivery.

Proportions of smoking frequency reduction (from daily to less than daily) and of smoking cessation, according to the moment of pregnancy (up to a month before, first, second, and third trimesters) were calculated by maternity. Gestational age was verified in prenatal cards and medical records, and it was estimated according to an algorithm, which gave priority to different sources of data in the following order: data of the last menstrual period (LMP), if compatible with the last ultrasound (USG) made before 20 weeks; USG, when LMP values were not compatible or ignored; and, at last, the neonatal clinical exam. The information was later grouped in trimesters of pregnancy (1^st^ trimester <14, 2nd trimester from 14-27, and 3rd trimester ≥ 28 weeks of gestation).

Statistical differences according to maternal characteristics and adequacy of prenatal care between maternities were evaluated by Mann-Whitney (ordinal variables), Pearson’s chi-square (nominal variables) test and ANOVA (continuous variables) tests. Prevalence rates and 95% confidence intervals (95% CI) were also calculated according to maternity. The association between smoking prevalence and categorical variables was tested based on Pearson’s chi-squared test. Chi-squared test of linear trend was used to evaluate gradient effect between smoking prevalence rate and ordinal categorical variables.

Variables associated with maternal smoking prevalence in univariable analysis (level of statistical significance below 0.20) were selected for multivariable analysis. Variable categories could be aggregated according to frequency distribution and cut points adopted in other studies. The dependent variable was *smoking at any moment starting one month before the pregnancy up to labor* (yes/no), and the independent variables selected in the previous stage (ordinal categorical variables). The variables with statistical significance level lower than 0.05 remained in the final model. Simple and multiple logistic regression models were conducted for each maternity. The data were analyzed by Statistical software SPSS®, version 17.

This study was approved by the Ethical review Board of Federal University of Rio de Janeiro. All women that participated in this study signed an informed consent term. For adolescents, a responsible person signed the term after adolescent signed an assent term.

## Results

From 1,744 singleton mothers eligible for the study, 549 were from maternity A and 1,195 were from maternity B. Missing information on maternal smoking was 16.6% (n = 90) and 1.6% (n = 19), so leaving 459 (MA) and 1,175 (MB) women in subsequent analysis. They were mostly of 20-34 years old, with partner, brown, with 8-11 years of study, with monthly family income between $88.47 and $331.75, multiparous, and with intermediate or inadequate prenatal care, in both maternities (Table 1). Except for partner, skin color, parity and adequacy of prenatal care, there were statistically significant differences between the maternities. The proportion of adolescents, low maternal educational level and low family income were higher in MA compared to MB.

**Table 1:** **Maternal and prenatal characteristics according to the public maternity**

**Maternal and prenatal characteristics**	**Maternity/City**				
	**A**		**B**		
	**Niterói**		**Rio de Janeiro**		
	**n**	**%**	**n**	**%**	**p-value**
Age group (years)^a^					0.02
10 – 19	142	30.9	292	24.9	-
20 – 34	279	60.8	769	65.4	-
≥35	38	8.3	114	9.7	-
Partner^b^					0.49
Absence	131	28.8	351	30.5	-
Presence	324	71.2	798	69.5	-
Skin color^b^					0.33
White	121	26.8	323	28.6	-
Brown	212	47.0	550	48.7	-
Black	118	26.2	256	22.7	-
Maternal Education Level (years of study)^a^					0.01
≤4	13	2.9	34	2.9	-
5-7	152	33.4	299	25.5	-
8-11	277	60.9	791	67,4	-
≥12	13	2.9	50	4.3	-
Family income per month (USD)^a^					0.06
<$88.47	96	23.2	228	20.9	-
$88.47 to $331.47	271	65.6	694	63.7	-
≥$331.47	46	11.1	167	15.3	-
Parity^b^					0.67
primiparous	185	40.3	487	41.4	-
multiparous	274	59.6	688	58.6	-
Adequacy of prenatal care^a^					0.10
Inadequate	148	35.7	411	37.9	-
Intermediate	168	40.5	465	42.9	-
Adequate	83	20.0	194	17.9	-
More than adequate	16	3.9	15	1.4	-

^a^Mann-Whitney test; ^b^Pearson’s chi-squared.

Note: The sums of the frequencies of the categories for each variable are not equal due to exclusion of records with missing information or information ignored in the analysis.

Smoking prevalence rates were high, 24.8% (n = 114; 95% CI: 21.1-29.0) and 17.9% (n = 210; 95% CI:15.8-20.1), respectively, in maternities A and B. Regardless of maternal characteristics, smoking prevalence were higher at MA, if compared to MB (except for higher income) (Table 2). Both maternities showed greater smoking prevalence among women aged 20-34 years, without partners, and multiparous. Regarding skin color, smoking was more prevalent among brown, at MA, and black at MB. An inversely proportional gradient between smoking prevalence and income, maternal education level and adequacy of prenatal care (Kotelchuck index), statistically significant (expect for income at maternity A) were observed. The association between smoking during pregnancy and maternal age, as continuous variable, did not present statistical significance for the ANOVA test (p-value < 0.05; data not shown).

**Table 2:** **Prevalence of maternal smoking during pregnancy according to maternal and prenatal characteristics and public maternity**

**Maternal and prenatal characteristics**	**Maternity/City**			
	**A - Niterói**		**B - Rio de Janeiro**	
	**Prev.** ^**a**^ **(%)**	**p-value**	**Prev.** ^**a**^ **(%)**	**p-value**
Age group (years)^b^		0.21		0.01
10 - 19	19.1	-	10.3	-
20 - 34	28.3	-	21.2	-
≥35	21.1	-	14.9	-
Partner^c^		0.32		0.22
absence	28.2	-	20.2	-
presence	23.8	-	17.2	-
Skin color^c^		0.40		<0.01
white	20.7	-	15.5	-
brown	27.4	-	15.1	-
black	24.6	-	24.6	-
Maternal Education Level (years of study)^b^		<0,01		<0.01
≥4	15.4	-	20.6	-
5-7	36.8	-	24.4	-
8-11	19.1	-	15.4	-
≥12	15.4	-	14.0	-
Family income per month (USD)^b^		0.14		0.04
<$88.47	27.1	-	22.8	-
$88.47 to $331.47	26.2	-	17.4	-
≥$331.47	13	-	13.2	-
Parity^c^		<0.01		<0.01
primiparous	11.9	-	11.1	-
multiparous	33.6	-	22.7	-
Adequacy of prenatal care^b^		<0.01		<0.01
Inadequate	31.1	-	20.4	-
Intermediate	21.4	-	15.9	-
Adequate	16.9	-	11.9	-
More than adequate	12.5	**-**	6.7	**-**
Total	24.8	-	17.9	-

^a^Prevalence; ^b^Chi-square Linear Trend; ^c^Pearson’s chi-square test.

Note: The sums of the frequencies of the categories for each variable are not equal due to exclusion of records with missing information or information ignored in the analysis.

Age, skin color, income, maternal educational level, parity, and adequacy of prenatal care were selected (p < 0.20) for multivariable analysis in both maternities. Education level and adequacy of prenatal care had their categories aggregated, as binary and three-category variables respectively.

Table 3 shows the unadjusted (OR_unadj_) and adjusted (OR_aj_) odds ratios for maternal, prenatal, and smoking characteristics during pregnancy. After multivariable analysis, low educational level and multiparity were positively associated to maternal smoking in both maternities. In maternity B model, black skin color and inadequate prenatal care also remained positively associated, while adolescence was negatively associated (Table 3).

**Table 3:** **Maternal and prenatal characteristics and their relation with smoking during pregnancy, according to public maternity**

**Maternal and prenatal characteristics**	**Maternity/City**					
	**A - Niterói**			**B - Rio de Janeiro**		
	**OR**_**unadj**_^**a**^	**OR**_**aj**_^**b**^	**95% CI**	**OR**_**unadj**_^**a**^	**OR**_**aj**_^**b**^	**95% CI**
Age group (years)						
10 - 19	0.59^c^	0.77	0.38;1.54	0.43^c^	0.44^c^	0.26;0.75
20 - 34	1	1		1	1	
≥35	0.68	0.60	0.23;1.53	0.65^d^	0.55^d^	0.29: 1.04
Partner						
absence	1,26	-	-	1,22	-	-
presence	1	-	-	1	-	-
Skin color						
white	1	1		1	1	
brown	1.45^d^	0.98	0.52;1.88	0.97	0.93	0.60;1.44
black	1.25	0.88	0.43;1.82	1.78^c^	1.71^c^	1.06;2.74
Maternal Education Level (years of study)						
<8	2.32^c^	2.14^c^	1.21;3.79	1.75^c^	1.61^c^	1.08;2.40
≥8	1	1		1	1	
Family income per month (USD)						
<$88.47	2,48^d^	1.12	0.38;3.27	1.95^c^	1.33	0.68;2.60
$88.47 to $331.47	2.36^d^	1.57	0.60;4.09	1.39^d^	1.18	0.67;2.10
≥$331.47	1	1		1	1	
Parity						
primiparous	1	1		1	1	
multiparous	3.75^c^	3.48^c^	1.78;6.81	2.35^c^	1.58^c^	1.03;2.44
Adequacy of prenatal care						
Inadequate	2.34^c^	1.80	0.89;3.64	1.98^c^	1.86^c^	1.07;3.25
Intermediate	1.42	1.09	0.53;2.22	1.46^d^	1.57	0.90;2.72
Adequate/More than adequate	1	1		1	1	

CI: Confidence Interval.

ORu_nadj_^a^:Unadjusted odds ratio; OR_aj_^b^:Adjusted odds ratio, ^c^p < 0.05 and ^d^p < 0.20.

Note: Logistic regression model (maternity A: age group, education, income, parity, adequacy of prenatal care; maternity B: age group, skin color, education, income, and parity, and adequacy of prenatal care).

Among women who stated they smoked one month before pregnancy onset, 104 and 183 with complete information, from MA and MB, respectively, were analyzed concerning continuity, reduction, or cessation of the smoking habits up to labor (Table 4).

**Table 4:** **Smoking maintenance, reduction and cessation in pregnancy, according to public maternity**

	**Maternity/City** ^**a**^	
**Maternal smoking**	**A Niterói**	**B Rio de Janeiro**
N^o^ of smokers before pregnancy onset^b^	104	183
Smoking Indicators (%)		
Maintenance of frequency	62.5	53.0
Reduced frequency in the 1^st^ trimester and kept	1.9	4.9
Reduced frequency in the 2^st^ trimester and kept	5.8	6.6
Reduced frequency in the 3^st^ trimester	1.9	0.5
Stopped in the 1st trimester	8.7	18.0
Stopped in the 2st trimester	13.5	11.5
Stopped in the 3st trimester	5.8	2.7
Reduced in the 1st trimester and stopped in the 2nd trimester	0.0	1.1
Reduced in the 2nd trimester and stopped in the 3rd trimester	0.0	1.6

^a^p value = 0.13 (Pearson chi square test).

^b^The study excluded records with missing or ignored information for any questions on smoking and 8 records stated the onset and maintenance of smoking during pregnancy.

Note: Reduction of smoking habit frequency = daily to less than daily.

More than half of smoker mothers kept smoking habits during pregnancy (Table 4). Maternity A showed not only greater prevalence of maternal smoking but it presented, overall, less smoking reduction and cessation than maternity B (Table 4). The reduction of smoking frequency was greater between the first and second trimesters of pregnancy in both maternities. Regarding cessation of smoking habits, the proportion was higher between the first and second trimesters of pregnancy at MA (13.5%), and between one month before pregnancy onset and the first trimester at MB (18%). The reduction and cessation of smoking habits were lower in the last trimester of pregnancy in both maternities. There was no reduction and subsequent cessation of smoking habits up to labor at MA. No statistical difference between maternities was observed (p value = 0.13).

## Discussion

Maternal smoking prevalence in this study, during any moment, from one month before pregnancy onset up to labor, was high. The comparability of these results with national and international studies is limited, mostly due to different measurements of smoking habits, regional variations, and trends of smoking habits in Brazil [1,6]. Women assisted by public prenatal care in six Brazilian capitals, in the nineties, had smoking prevalence in the second trimester of pregnancy ranging from 7.2% to 31.9%. For Rio de Janeiro city, the prevalence was 18% [16]. In a multicenter survey at primary care units in Latin America (2004-2005), Brazilian smoking prevalence during pregnancy was only 6.1% [17]. A study conducted by Levy et al. [3] used estimates from surveys and from some individual studies, and calculated, for 2008, a 7.7% smoking prevalence among pregnant women in Brazil. However, values obtained in a southern Brazilian city (23%) in 2007[4], were closer to our results. Heterogeneity in smoking prevalence observed in Brazil was reproduced in two cities located in the state of Rio de Janeiro.

Smoking prevalence in high-income countries during pregnancy ranged from 6% to 22% [18]. The United Kingdom [19] presented a 26% maternal smoking prevalence during pregnancy in 2010, and Canada presented 10.5% in 2005 and 2006 [20].

Smoking frequency during pregnancy, obtained in a self-reported manner, may be underestimated if compared to the estimates obtained by measuring biochemical markers, such as cotinine levels [7]. On the other hand, classification errors may have been minimized with the interviews after delivery and the validation of the information every each trimester [12]. Therefore, even this high prevalence of smoking in pregnancy at the maternities in the cities of Rio de Janeiro and Niterói may be underestimated.

It is worth mentioning that in addition to validation problems, the restriction to liveborn singleton mothers and the definition of smoking, constrained to active smoking, may have interfered in the magnitude of the smoking prevalence during pregnancy of these maternities. The inclusion of women hospitalized due to abortion or stillbirth, which are outcomes associated with smoking during pregnancy [3], would tend to raise the prevalence, as well as the measuring of the exposure to secondhand smoking.

Associations among smoking during pregnancy, black skin color [21], low education level [7,13,16], multiparity, and inadequacy of prenatal care [7,13] reported in scientific literature were also observed in this study. Educational level and multiparity were found in both maternities and could be generalized for similar populations. Smoking prevalence during pregnancy was related to social disparities with higher smoking prevalence in less educated women. Mothers assisted at MA showed lower educational level compared to those assisted at MB, which may justify the higher prevalence of smoking in MA.

More than half of the pregnant smokers kept the same smoking frequency during pregnancy up to labor. This outcome is worrisome if compared to values of smoking continuance in other studies, such as 12% in the United Kingdom in 2010 [19] and 26.2% in 15 Europeans countries, between 2011 and 2012 [22]. The highest percentages of smoking frequency reduction between the first and second trimesters of pregnancy may occur, partially, due to late acknowledgement of pregnancy. Smoking cessation was more frequent before and in the pregnancy onset, which is a positive result, considering significant benefits for the fetal development with the early cessation of the smoking habit [10]. There are not many studies on smoking cessation in pregnant women in Brazil. In the southern Brazilian city of Pelotas, from 1989 to 1990, smoking cessation of pregnant women in the second trimester of pregnancy was 35.6% and it was associated with higher education levels and nonsmoker partner [23]. In a study of 486 pregnant women at health care centers in Rio de Janeiro and Niterói cities (2003/2004), smoking prevalence was 21%. A low score of smoking dependence was identified, suggesting susceptibility to cognitive-behavioral cessation measures [24]. Out of the total number of women, 36% stopped smoking in the first trimester, with no specific interventions, probably because of concern for the baby. There was no support for smoking cessation in the four health care unities investigated [24].

Smoking cessation during pregnancy may be considered a “temporary abstinence”, especially for women under social economic disadvantage. Women that stopped smoking during pregnancy showed greater risk to restart the habit after the childbirth [18]. Thus, health care professionals must be proactive and trained to deal with women who desire to become pregnant, who are pregnant, and under postpartum period, always looking for opportunities to promote cessation of smoking habits. Population and individual strategies to prevent and control the use of tobacco must have actions specific for women, alerting for the negative consequences for their own health, as well for the baby’s health.

The effectiveness of population and individual strategies to prevent onset and promote smoking cessation for pregnant women and for those in the reproductive age has been evaluated [12,25,26]. Clinical strategies, advising, and pharmacological treatments in high-income countries showed good results, although there is controversy concerning safety and efficacy of the latter strategy in pregnant women. In seven middle and low-income cities of Latin America countries, including Pelotas, southern Brazilian city, only educative and psychological support actions were not effective [25]. The pooled effects were similar in interventions provided for women with predominantly low socioeconomic status, compared to other women [26].

Smoking control measures adopted by Brazil since 1986 have contributed for its reduction, although in an unequal manner [1]. The national strategy for smoking tendency monitoring in adults [2], named Surveillance System of Risk Factors and Protection against Chronic Diseases by Phone Survey (VIGITEL), should also identify pregnant women during the phone surveys to monitor this population group and to propose specific interventions.

## Conclusion

Smoking prevalence during pregnancy was higher for multiparous and less educated women. Population and individual strategies for smoking prevention and control must include actions specific for women, especially during the reproductive period.

## Abbreviations

ANOVA: Analysis of Variance
CNPq: National Council for Scientific and Technological Development
LMP: date of last menstrual period
FAPESP: Foundation to Support research in the state of São Paulo
FAPERJ: Carlos Chagas Filho Foundation for Research Support of the State of Rio de Janeiro
FIOCRUZ: Oswaldo Cruz Foundation
95% CI: Confidence Interval of 95%
IESC: Institute for Studies in Public Health
IFF: Fernandes Figueira Institute
ISC: Institute of Community Health
MA: Maternity A
MB: Maternity B
MS: Ministry of Health
OR: Odds ratio
PET: Education Program for Health at Work
PPSUS: Research Program for the National Health System
SP: São Paulo
SPSS: Statistical Package for Social Sciences
SUS: National Health System
VIGITEL: Surveillance System of Risk Factors and Protective against Chronic Diseases by Telephone Survey
UFF: Federal Fluminense University
UFRJ: Federal University of Rio de Janeiro
USD: US dollar
USP: University of São Paulo
USG: ultrasound

## References

[1] Szklo AS, Almeida LM, Figueiredo VC, Autran M, Malta D, Caixeta R (2012). A snapshot of the striking decrease in cigarette smoking prevalence in Brazil between 1989 and 2008. Prev Med.

[2] Ministério da Saúde (2013). Secretaria de Vigilância em Saúde Departamento de Vigilância de Doenças e Agravos Não Transmissíveis e Promoção da Saúde. VIGITEL 2012: Vigilância de fatores de risco e proteção para doenças crônicas por inquérito telefônico.

[3] Levy D, Jiang M, Szklo A, de Almeida LM, Autran M, Bloch M (2013). Smoking and adverse maternal and child health outcomes in Brazil. Nicotine Tob Res.

[4] Zhang L, González-Chica DA, Cesar JA, Mendoza-Sassi RA, Beskow B, Larentis N (2011). Tabagismo materno durante a gestação e medidas antropométricas do recém-nascido: um estudo de base populacional no extremo sul do Brasil. Cad Saude Publica.

[5] Lombardi EMS, Prado GF, Santos UP, Fernandes FLA (2011). O tabagismo e a mulher: Riscos, impactos e desafios. J Bras Pneumol.

[6] Monteiro CA, Cavalcante TM, Moura EC, Claro RM, Szwarcwald CL (2007). Population-based evidence of a strong decline in the prevalence of smokers in Brazil (1989-2003). Bull World Health Organ.

[7] Cnattingius S (2004). The epidemiology of smoking during pregnancy: smoking prevalence, maternal characteristics, and pregnancy outcomes. Nicotine Tob Res.

[8] Rasmussen M, Heitmann BL, Tønnesen H (2013). Effectiveness of the Gold Standard Programmes (GSP) for smoking cessation in pregnant and non-pregnant women. Int J Environ Res Public Health.

[9] Hinkle S, Albert P, Mendola P, Sjaarda L, Boghossian N, Yeung E (2014). Differences in risk factors for incident and recurrent small-for-gestational-age birthweight: a hospital-based cohort study. BJOG.

[10] Sclowitz IK, Santos IS, Domingues MR, Matijasevich A, Barros AJ (2013). Maternal smoking in successive pregnancies and recurrence of low birthweight: the 2004 Pelotas birth cohort study, Brazil. Cad Saude Publica.

[11] Horta BL, Gigante DP, Silveira VMF, Oliveira I, Victora CG (2011). Maternal smoking during pregnancy and risk factors for cardiovascular disease in adulthood. Atherosclerosis.

[12] Lumley J, Chamberlain C, Dowswell T, Oliver S, Oakley L, Watson L (2009). Interventions for promoting smoking cessation during pregnancy. Cochrane Database Syst Rev.

[13] Mohsin M, Bauman AE (2005). Socio-demographic factors associated with smoking and smoking cessation among 426,344 pregnant women in New South Wales, Australia. BMC Public Health.

[14] Canada. Statistics Canada. National Longitudinal Survey of Children and Youth, 2008/2009. Available: http://www23.statcan.gc.ca/imdb-bmdi/instrument/4450_Q2_V7-eng.pdf [Accessed on Apr 16^th^ 2015].

[15] Kotelchuck M (1994). An evaluation of the Kessner Adequacy of Prenatal Care Index and a Proposed adequacy of Prenatal Care Utilization Index. Am J Public Health.

[16] Kroeff LR, Mengue SS, Schmidt MI, Duncan BB, Favaretto AL, Nucci LB (2004). Fatores associados ao fumo em gestantes avaliadas em cidades brasileiras. Rev Saude Publica.

[17] Bloch M, Althabe F, Onyamboko M, Kaseba-Sata C, Castilla EE, Freire S (2008). Tobacco use and secondhand smoke exposure during pregnancy: an investigative survey of women in 9 developing nations. Am J Public Health.

[18] Nichter M, Greaves L, Bloch M, Paglia M, Scarinci I, Tolosa JE (2010). Tobacco use and secondhand smoke exposure during pregnancy in low- and middle-income countries: the need for social and cultural research. Acta Obstet Gynecol Scand.

[19] Hardy B, Szatkowski L, Tata LJ, Coleman T, Dhalwani NN (2014). Smoking cessation advice recorded during pregnancy in United Kingdom primary care. BMC Fam Pract.

[20] Al-Sahab B, Saqib M, Hauser G, Tamim H (2010). Prevalence of smoking during pregnancy and associated risk factors among Canadian women: a national survey. BMC Pregnancy Childbirth.

[21] Tabb KM, Huang H, Meneses PR, Silva GA, Chan Y, Faisal-Cury A (2014). Ethnic differences in tobacco use during pregnancy: findings from a primary care sample in São Paulo, Brazil. Ethn Health.

[22] Smedberg J, Lupattelli A, Mårdby AC, Nordeng H (2014). Characteristics of women who continue smoking during pregnancy: a cross-sectional study of pregnant women and new mothers in 15 European countries. BMC Pregnancy Childbirth.

[23] Halal IS, Victora CG, Barros FC (1993). Determinantes do hábito de fumar e de seu abandono durante a gestação em localidade urbana na região sul do Brasil. Rev Saude Publica.

[24] Reis LG, da Silva CJ, Trindade A, Abrahao M, da Silva VA (2008). Women who smoke and stop during pregnancy: Who are they?. Rev Bras Saude Matern Infant.

[25] Oncken CA, Dietz PM, Tong VT, Belizán JM, Tolosa JE, Berhella V (2010). Prenatal tobacco prevention and cessation interventions for women inlow- and middle-income countries. Acta Obstet Gynecol Scand.

[26] Chamberlain C, O’Mara-Eves A, Oliver S, Caird JR, Perlen SM, Eades S (2013). Psychosocial interventions for supporting women to stop smoking in pregnancy. Cochrane Database Syst Rev.

